# Development and Evaluation from Laboratory to Field Trial of a Dual-Purpose Fracturing Nanofluid: Inhibition of Associated Formation Damage and Increasing Heavy Crude Oil Mobility

**DOI:** 10.3390/nano12132195

**Published:** 2022-06-26

**Authors:** María A. Giraldo, Richard D. Zabala, Jorge I. Bahamón, Juan M. Ulloa, José M. Usurriaga, José C. Cárdenas, Camilo Mazo, Juan D. Guzmán, Sergio H. Lopera, Camilo A. Franco, Farid B. Cortés

**Affiliations:** 1 Grupo de Investigación Fenómenos de Superficie—Michael Polanyi, Departamento de Procesos y Energía, Facultad de Minas, Universidad Nacional de Colombia—Sede Medellín, Medellín 050034, Colombia; maragiraldomun@unal.edu.co (M.A.G.); cmazos@unal.edu.co (C.M.); jdguzmanc@unal.edu.co (J.D.G.); 2 Departamento de Tecnologías de Producción, Ecopetrol S.A., Bogotá D.C. 111311, Colombia; richard.zabala@ecopetrol.com.co (R.D.Z.); jorge.bahamon@ecopetrol.com.co (J.I.B.); 3 Coordinación de Ingeniería, Gerencia Castilla, Ecopetrol S.A., Castilla La Nueva 507041, Colombia; juan.ulloa@ecopetrol.com.co; 4 Instituto Colombiano del Petróleo-ICP, Ecopetrol S.A., Piedecuesta 681011, Colombia; jose.usuriaga@ecopetrol.com.co (J.M.U.); jose.cardenasmo@ecopetrol.com.co (J.C.C.); 5 Grupo de Investigación Yacimientos de Hidrocarburos, Departamento de Procesos y Energía, Facultad de Minas, Universidad Nacional de Colombia—Sede Medellín, Medellín 050034, Colombia; shlopera@unal.edu.co

**Keywords:** field test, nanofluid, fracturing fluid, heavy crude oil, mobility, formation damage, rheological behavior

## Abstract

This study aims to develop and evaluate fracturing nanofluids from the laboratory to the field trial with the dual purpose of increasing heavy crude oil mobility and reducing formation damage caused by the remaining fracturing fluid (FF). Two fumed silica nanoparticles of different sizes, and alumina nanoparticles were modified on the surface through basic and acidic treatments. The nanoparticles were characterized by transmission electron microscopy, dynamic light scattering, zeta potential and total acidity. The rheological behavior of the linear gel and the heavy crude oil after adding different chemical nature nanoparticles were measured at two concentrations of 100 and 1000 mg/L. Also, the contact angle assessed the alteration of the rock wettability. The nanoparticle with better performance was the raw fumed silica of 7 nm at 1000 mg/L. These were employed to prepare a fracturing nanofluid from a commercial FF. Both fluids were evaluated through their rheological behavior as a function of time at high pressure following the API RP39 test, and spontaneous imbibition tests were carried out to assess the FF’s capacity to modify the wettability of the porous media. It was possible to conclude that the inclusion of 7 nm commercial silica nanoparticles allowed obtaining a reduction of 10 and 20% in the two breakers used in the commercial fracture fluid formulation without altering the rheological properties of the system. Displacement tests were also performed on proppant and rock samples at reservoir conditions of overburden and pore pressures of 3200 and 1200 psi, respectively, while the temperature was set at 77 °C and the flow rate at 0.3 cm^3^/min. According to the effective oil permeability, a decrease of 31% in the damage was obtained. Based on these results, the fracturing nanofluid was selected and used in the first worldwide field application in a Colombian oil field with a basic sediment and water (BSW%) of 100 and without oil production. After two weeks of the hydraulic fracture operation, crude oil was produced. Finally, one year after this work, crude oil viscosity and BSW% kept showing reductions near 75% and 33%, respectively; and having passed two years, the cumulative incremental oil production is around 120,000 barrels.

## 1. Introduction

Nearly 56% of the non-conventional oil, representing a little more than half of the total recoverable oil resources, corresponds to heavy, extra-heavy and bitumen [1]. However, this kind of hydrocarbons produces technical problems associated with their typical high viscosity values, making their mobility difficult in the reservoir and, hence, their production, transport and refining due to their chemical nature [2,3,4]. In this sense, some techniques have been developed and probed to reduce the crude oil viscosity and can be classified as non-thermal and thermal based on improved oil recovery (IOR) or enhanced oil recovery (EOR) process. For example, in the first group, the injection of diluents into the reservoir generally lights hydrocarbons such as light crude oil and naphtha [5,6,7]. The thermal applications consist of techniques that generate in-situ upgrading of crude oil [8,9] or injecting hot fluids such water steams to the reservoir without generating chemical reactions [10,11]. In the first case, the viscosity reduction is permanent, while in the second is temporary and depends on the hydrocarbon temperature [7]. Other techniques point to generating wettability alterations that improve crude oil mobility inside the porous media, as the injection of surfactants [12,13]. In this sense, hydraulic fracturing operation also has been used to stimulate several wells producing heavy oil generating, in a particular Colombian oil field, increments in all the 43 wells intervened with this technique [14]. However, this technique has been associated with inherent damage due to the low percentage of fracturing fluid recovered in the flowback, being typically lower than 50% [15], and causing damage rate in permeabilities of even almost 98% [16].

On the other hand, nanotechnology has been used in the oil and gas industry, along its productive chain, to enhance different processes and solve specific problems more efficiently than other technologies [17]. All this is due to the unique characteristics of nanomaterials, such as high surface area/volume ratio, small size, and the possibility of adjusting their properties to the technical and operational requirements [18]. So, nanotechnology has been implemented to improve crude oil properties as can be seen in Vakhim et al. research [19]. Authors found that employing nanosized magnetite, in presence of microwave, can reduce the percentage of resins and asphaltenes in crude oil. This situation can lead to obtaining crude oil of higher quality, with lower viscosities. Precisely, nanotechnology has been also used to reduce the viscosity of heavy crude oils, as Taborda et al. [7] studied the effect of nanoparticles/nanofluids on heavy crude rheology oil. They found viscosity reductions of up to 90% compared to the system without nanoparticles at reservoir conditions at the laboratory [7]. In this work, the authors also demonstrate the capacity of nanoparticles to increase the recovery in the coreflooding test, obtaining 16% more oil production capacity. This kind of application of nanotechnology has also been tested in the field by Zabala et al. [20], who use nanofluids to improve the mobility ratio in heavy oil fields, finding increments of up to 280 BOPD with a penetration radius of only 3 ft, besides BSW reduction of 11%. The skin damage was also reduced by about 73%, while the crude oil viscosity fell 47% in the first 30 days of production, confirming the ability of nanofluids to improve the mobility conditions of fluids in the reservoir. Precisely, one of the main factors determining these conditions is related to the wettability of the porous media [21]. In this sense, authors like Giraldo et al. [22] developed studies about using alumina nanoparticles to turn the preferential oil wettability of the evaluated porous media into water wettability. Similarly, Franco et al. [23], Betancur et al. [24] and Zhang et al. [25] also demonstrated significant improvements in wettability for porous media of heavy crude oil reservoirs using nanofluids of different chemical natures.

The use of nanotechnology in the hydraulic fracturing process has been reported in several studies [26,27,28,29,30]. For example, Lafitte et al. [30] applied nano-crosslinkers of 15 nm to decrease the polymer content in the fracture fluids, diminishing the amount of crosslinker and polymer to 20 times lower and 10%, respectively. Similarly, Lian et al. [26] found polymer reductions of up to 48%, obtaining a fluid capable of withstanding high temperatures (350–400 °C). The addition of nanoparticles to fracturing fluids has been mainly implemented to improve fluid conditions by reducing the additive content like there were done by Guzmán et al. [27]. They found that adding nanoparticles propitiate reduction in 33% of the methanol content in a commercial FF led to stimulating a water-sensitive formation and caused the reduction of the formation damages associated with this kind of fluids [27]. Another notable result of this work was improving the rock’s wettability conditions, reducing the water’s relative permeability by up to 83%. It is worth mentioning that in the scientific literature there is no reported usage of fracturing fluid with dual-purpose that looks for the reduction of the crude oil viscosity and the formation damage associated with the remaining fluid in the reservoir. Also, there is no evidence of applying a fluid of these characteristics in a field trial.

Therefore, the present study aims to develop and evaluate dual-purpose fracturing nanofluids (nanoFF) that allow increasing the mobility of heavy crude oil in porous media by three mechanisms: the changes in surface wettability, crude oil viscosity reduction, and reduce the formation damage associated. To achieve this, nanoparticles of different chemical nature (silica gel and alumina) were superficially modified and characterized by their size and morphology, surface area and acidity, and, finally, the zeta potential, determining their stability in the FF and how it affects its properties. Static and dynamic tests assessed the capacity of the nanoFFs to improve the wettability of the porous media and reduce the heavy oil viscosity, both of them improving the crude oil mobility. The static test includes rheological experiments carried to the heavy crude oil, the linear gel (LG), and the commercial FF in the absence and presence of nanoparticles. The evaluation of wettability alteration and the construction of the equilibrium adsorption isotherm were also included. The dynamic evaluation was carried out through core-flooding tests made with the original fracturing fluid and the nanoFF in the proppant porous media, a carbolite-based synthetic core, and in the reservoir’s original core.

Finally, an application in a Colombian field was performed, looking for improving the BSW% of the intervened well and the reduction in the crude oil viscosity, and both sustained for more than a year since the field trial.

## 2. Materials and Methods

### 2.1. Materials and Chemicals

Two nanoparticles of silica and alumina were chosen. A commercial SiO_2_ nanoparticles (Sigma-Aldrich, St. Louis, MO, USA) with different sizes were evaluated. These were named based on the mean particle sizes Si07 (silica of 7 nm) and Si200 (silica of 200 nm). Also, Al_2_O_3_ (Al) nanoparticles were employed (Petroraza S.A.S, Sabaneta, Colombia). HCl (37%, Sigma-Aldrich, San Luis, USA), NH_4_OH (30%, J.T. Baker, Phillipsburg, USA), and deionized water were used in the surface modification processes [31]. In surface acidity measurements, a gas mixture of NH_3_/He (10/90) and He (99.9%) was used (Linde, Medellín, Colombia).

A commercial water-based fracturing fluid (FF) was used with carboxymethyl hydroxypropyl gum guar as a polymer and metaborate as a crosslinker. However, it is remark to highlight the presence of a zeta potential modifier (ZPM), a compound used to agglomerate proppant in the reservoir once the hydraulic fracture is closed, preventing its return during the flowback stage. N_2_ (Linde, Medellín, Colombia) was employed for pressuring the system in the process of FF breaking off at laboratory scale. A Colombian heavy crude oil (HO) sample from Llanos Basin near Villavicencio City with 11.6° API and 90,000 cP of viscosity at a shear rate of 10 s^−1^ at 25 °C was used for carrying out the tests. Its mass fractions of saturates, aromatics, resins and asphaltenes (SARA) were 18.9%, 31.7%, 32.5% and 16.9%, respectively.

For the static tests related to alteration of porous media wettability, synthetic porous media were made composed of Ottawa sand (30/50 U.S. mesh, Minercol, Colombia). Toluene (99%, Sigma-Aldrich, St. Louis, MO, USA) and ethanol (99.9%, Sigma-Aldrich, St. Louis, MO, USA) were employed to wash any impurities in the samples. n-heptane (99%, Panreac, Darmstadt, Germany) was used for aging of rock samples.

For the coreflooding tests, a core provided by Ecopetrol SA (Castilla La Nueva, Colombia) was used, extracted from the formation that produced the extra-heavy crude oil used in this investigation. A carbolite porous medium of 20–40 of size was also used. Table 1 summarizes the properties of both porous media.

### 2.2. Methods

**Figure 1:**
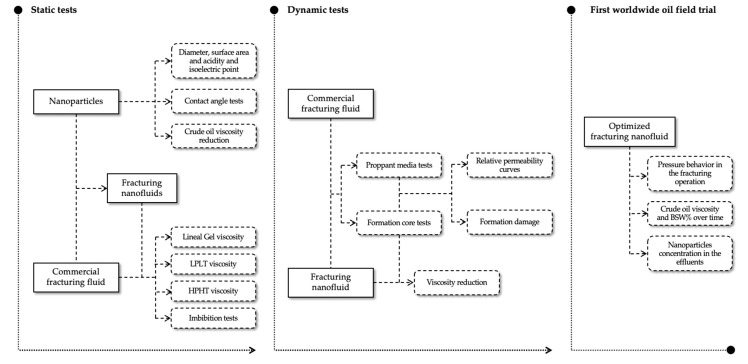
Experimental workflow for developing and testing a fracturing nanofluid from laboratory test to oil field trial.

This section presents all the experiments and tests carried out to develop a fracturing nanofluid to improve the crude oil mobility inside the porous media. In this sense, Figure 1 shows the experimental workflow beginning in the static tests, passing through the dynamic tests and ending in the first worldwide field trial of this kind of fluid.

#### 2.2.1. Nanoparticle Surface Modification

Surface modification of two nanoparticles was developed based on our previous works [24]. For obtaining a higher surface acidity, nanoparticles were introduced in deionized water at a 5 g/L concentration, and the pH was adjusted to a value of 2 with a 3% *v*/*v* HCl aqueous solution. The system was sonicated for 2 h and stirred for 12 h, at 300 rpm, all at 25 °C. Finally, the modified nanoparticles were dried for 20 h at 120 °C. The same process was repeated to obtain nanoparticles of a basic surface by adjusting the system’s pH at 13 with a 3% *v*/*v* NH_4_OH aqueous solution. The nomenclature used for differentiating the new nanoparticles was Si07A, Si200A and Al-A for acidified surfaces; and Si07B, Si200B and Al-B for basified surfaces.

#### 2.2.2. Nanoparticle Characterization

Different characterization techniques were used to determine the properties of the nanoparticles. Their sizes and morphologies were calculated using a high-resolution transmission electron microscopy (HR-TEM) with a Tecnai G2 F20 microscope (FEI, Hillsboro, OR, USA). The method proposed by Brunauer-Emmett-Teller [32] was used to measure the surface area (S_BET_) through N_2_ physisorption at −196 °C with an Autosorb-1 (Quantachrome Instruments, Boynton Beach, MIA, USA). A ChemBet 3000 (Quantachrome Instruments, Boynton Beach, MIA, USA) with a thermal conductivity detector was used to measure surface acidity. These measurements were carried out with NH_3_ temperature-programmed desorption (TPD) [31]. Finally, to determine the stability of the nanoparticles at different pH conditions, the zeta potential analysis was carried out using a zetasizer analyzer nanoplus-3 from Micromeritics (Norcross, GA, USA).

#### 2.2.3. Preparation of the Fracturing Fluids

A commercial fracturing fluid for high permeability reservoirs was used. It was prepared in nine stages, each adding a different additive that fulfills a specific function, as shown in Table 2. The order was: bactericide, clay stabilizer, guar gum-based polymer. At this moment, there was needed to wait 20 min to achieve the total polymer hydration guaranteeing the proper viscosification of the fluid. At the end of this stage, it is considered that the FF is in the linear gel phase (LG). After that, there was also added surfactant, ZPM, pH controller, two types of breakers (a delayed breaker and a peroxide breaker), and finally, the metaborate crosslinker. All the additives were mixed at 600 rpm. After adding the polymer, immediately there were also added the nanoparticles for those FF that were meant to contain them. The process continues just as previously described.

#### 2.2.4. Rheological Experiments

Three different sets of rheological experiments were carried out depending on their purpose. Two of them were performed at atmospheric pressure and the last one at high pressure, just as follows:

- The first kind of test related to low-pressure conditions was performed using a Kinexus Pro^+^ rotational rheometer (Malvern Instruments, Worcestershire, UK) with a Peltier plate for temperature control. At first, the viscosity changes in the HO induced by the presence of the nanoparticles were evaluated. In this case, a low (100 mg/L) and a high (1000 mg/L) concentration of these were mixed with the crude oil by stirring at 500 rpm for 30 min until homogenization [7]. The measures were made using a plate-plate geometry at a gap of 300 μm at 25 °C in a shear rate range of 1 to 100 s^−1^.
- Other tests at low pressure were carried out to determine if nanomaterials tend to aggregate in the presence of ZPM, as the proppant does. For this purpose, the LG was tested in the presence and absence of ZPM with or without the different nanoparticles at a concentration of 1000 mg/L. This set of rheological measurements was made with the geometry of the solid cylinders at 25 °C at a shear rate between 1 and 100 s^−1^. As mentioned above, the FF viscosity is one of the main properties of this kind of fluid due to its importance in placing the proppant in the open fracture according to the plan [27,33]. Hence, rheological measures were made to the original FF and samples of this when adding 100 and 1000 mg/L of the different nanoparticles. The solid cylinders geometry was used at the same temperature and fixed shear rates of 25, 50, 75 and 100 s^−1^. The purpose of this test is to emulate at low pressures and temperatures the first effort of the API RP39 test [34], the standard for evaluating the rheological properties of the fracturing fluids.
- Precisely, this is the third type of test, and a high pressure—high temperature Chandler 5550 viscometer (Chandler Engineering, Broken Arrow, USA) was used at the expected reservoir and fracking conditions 77 °C and 5500 psi. This procedure allows following the viscosity of the evaluated fracturing fluids when arming and through their rupture process under the action of shear rates of 25, 50, 75 and 100 s^−1^.

#### 2.2.5. Wettability Alteration

Qualitative water angle measures were developed as the first evaluation of nanoparticles as altering wettability agents of the porous media. This process involved making synthetic porous media of Ottawa sand with 4.6 cm of diameter and 2.0 cm of length. Oil wettability restoration was induced by aging the prepared cores into crude oil, as described in previous studies [22]. Then, the samples were soaked in different aqueous dispersions of nanoparticles at concentrations of 100 and 1000 mg/L for 24 h at reservoir temperature of 77 °C for 24 h. After, the plugs were dried at 40 °C for 12 h. Later, a droplet of water was placed over the surface of each sample at 25 °C and measures of contact angle were made.

Imbibition tests were also carried out to evaluate the FF’s capacity to modify the wettability of the porous media in the absence and presence of nanoparticles. The samples were prepared by pressurizing the synthetic plug samples with evaluated FF at 50 psi and 77 °C for 16 h, guaranteeing the rupture of these fluids. After a drying process at 40 °C for 12 h, the air-saturated samples were placed in water while their weights were measured using an electronic scale. The ratio between the weight and the maximum weight achieved at the equilibrium time was reported as a function of time [22].

#### 2.2.6. Equilibrium Adsorption Isotherms of Polymer over the Rock Surface

Adsorption of the guar gum over the rock surface was evaluated through batch adsorption experiments. Solutions with initial concentrations of 1000, 3000, 5000, 7000 and 9300 mg/L of guar gum-based polymer were prepared, the last corresponding to the equivalent of the used in the formulation of the fracturing fluid. A fixed mass of nanoparticles was added, keeping their concentration at 1000 mg/L [35]. The order for adding the components was defined attending the abovementioned procedure for the preparation of the FF, and it was equivalent to Method III described by Giraldo et al. [36]. The different mixtures were stirred at 200 rpm at 25 °C for 48 h seeking to ensure the adsorption equilibrium. For all samples, the nanoparticles containing adsorbed polymer were separated from the mixture by centrifugation for 2 h at 4500 rpm using a Hermle Z 306 Universal Centrifuge (Labnet, NJ, USA). Then, the remaining humidity was removed using a closed system with cesium fluoride, CsF [37]. The amount of adsorbed polymer *q* (mg/L) was determined by mass balance using thermogravimetric analyses (TGA) under an air atmosphere varying between 25 and 800 °C at a fixed heating rate of 5 °C/min with an airflow of 100 mL/min. All this was made using a Q50 thermogravimetric analyzer from TA Instruments (New Castle, USA) [36,38].

#### 2.2.7. Core-Flooding Tests

Two different types of core-flooding tests were performed depending on the used porous medium: (i) a carbolite-based synthetic core representing the proppant media, and (ii) an original core of the reservoir of interest. The experimental setup and the followed procedure were detailed in a previous study [27]. Tests were made with the porous media mentioned in Table 1 under the particular conditions of the reservoir, such as overburden and pore pressures of 3200 and 1200 psi, respectively. The temperature was set at 77 °C and the flow rate at 0.3 cm^3^/min.

The test began with constructing the base curves when the formation brine was injected, and the absolute permeability (Kabs) was measured. Then, crude oil was injected, and the oil effective permeability (ko) was measured. Subsequently, brine was injected again to construct the water (krw) and oil relative permeability (kro) curves, using the JBN method [39,40]. Then, there was injected the fracture fluid in different ways, depending on the evaluated porous media: (i) in the carbolite pack, this was soaked with the disarmed FF for 24 h at reservoir conditions, and then oil and brine were injected into the production direction to construct the relative permeability curves; (ii) on the other hand, a specially designed core holder was used for the tests carried out in the reservoir cores [27]. This setup allowed the contact between these porous media and the armed fracturing fluids until their rupture at the fracturing pressure of 4500 psi. In this case, oil and brine were also injected in the production direction to construct the relative permeability curves. For both types of tests, the percentage of crude oil recovered was determined by the ratio between the recovered oil and the mobile oil. It is essential to mention that produced crude oil samples were collected each 0.5 porous volume after its injection in the final stage. The rheological properties of these samples were measured following the previously described method.

#### 2.2.8. Field Trial in a Colombian Oilfield

An application of the developed fracturing nanofluid was carried out in a Colombian oilfield, located in the Llanos Basin 50 km to the SE of the Villavicencio City in the Meta department in Colombia [14,20]. For this, a formation with petrophysical porosity values between 8 and 17.4% and permeability ranges from 254 to 1424 mD was selected [14]. The initial water saturation of this formation was originally around 15%, and the typically produced HO has an API gravity near to 13°, additionally, mobility ratio is 47. The reservoir temperature is estimated at 87.7 °C [14,20]. In one well of this field was made a fracking operation that used 126 bbls of the proposed fracturing nanofluid (nanoFF) to initiate the fracture, reaching pressures above 4500 psi.

The preflux fluid loaded with nanotechnology as a conditioner was pumped during the diagnostic pumping stages, a soak time to enhance the adsorption of the upcoming nano stage was promoted and a mixture of diesel and xylene (DIX) was injected to avoid any effect associated with the presence of organic deposits. Then, the synthetized nanoparticles to enhance mobility of the oil through interaction with the porous media was loaded in the first portion of the pad stage. 121,600 lb of proppant were placed and 486 bbls of the designed nanofluid was pumped.

### 2.3. Modelling

#### 2.3.1. Rheological Model

Equation (1) describes the Cross rheological model used to fit the experimental measures of viscosity (μ) at different shear rates (γ), in cP and s^−1^, respectively [7]. The flow behavior index *m* is related to the behavior of the fluid and if it has a more or less Newtonian behavior. For values below 1, the fluid is considered pseudo-plastic [7,41]. The viscosity at zero shear rate ($\mu_{0,\gamma }$) and at an infinite shear rate ($\mu_{\infty ,\gamma }$), both in cP, are asymptotic and indicate the fluid’s behavior when subjected to conditions near zero and infinite stresses. The $\alpha_{c}$ The parameter named characteristic relaxation time is related to the time required for the fluid to respond to a perturbation caused by agitation, and for a Newtonian fluid has a value of 0. The root-mean-square error (RMSE%) was used to estimate the goodness of fit with the Solver feature in Excel Professional Plus 2019 [42,43]. There was also used for the adsorption model.

(1) $$\mu =\mu_{\infty ,\gamma }+\frac{\mu_{0,\gamma }-\mu_{\infty ,\gamma }}{1+{\left(\alpha_{c}\gamma \right)}^{m}}$$

The degree of viscosity reduction (*DVR%*), in percentage, was calculated from Equation (2) where $\mu_{rev}$ and $\mu_{nanot}$ are the reference and the after-nanotechnology inclusion values for viscosity, in cP, measured at 10 s^−1^, respectively.

(2) $$DVR\%=\frac{\left(\mu_{rev}-\mu_{nanot}\right)}{\mu_{rev}}\times 100$$

#### 2.3.2. SLE Model

The SLE model described by Equations (3)–(5) is based on a theoretical explanation of the adsorption of self-associated molecules like polymers on solid surfaces [44], based on the theory of association proposed by Talu and Maunier [45]. The parameter *q* (mg/g) is the amount of polymer adsorbed, *qm* (mg/g) is the nanoparticle maximum adsorption capacity of the polymer, and *C_E_* is the equilibrium concentration of polymers in the solution.

(3) $$C_{E}=\frac{\Psi H}{1-K\Psi }e^{\left(\frac{\Psi }{q_{m}}\right)}$$

(4) $$\Psi =\frac{-1+\sqrt{1+4K\cdot \xi }}{2K}$$

(5) $$\xi =\left(\frac{q_{m}\cdot q}{q_{m}-q}\right)$$

Finally, *K* (g/g) is the reaction constant related to the degree of association of the polymer molecules in the surface of the nanoparticles, and *H* (mg/g) is Henry’s law constant linked to the preference of the polymer molecules for being in the liquid phase or the adsorbed phase [44]. 

## 3. Results

The workflow of this research, presented in Figure 1, began with the surface modification and characterization of different nanoparticles’ chemical natures and sizes, like silica of 7 nm, silica of 200 nm and alumina. These were also evaluated by their capacity of improving the crude oil mobility in the porous media through its viscosity reductions and surface wettability modifications. The work continues evaluating the impact of adding nanoparticles into a commercial fracturing fluid and its rheological behavior, especially in the presence of the zeta potential modifier. All these assessments allowed to optimize the formulation of the commercial FF in the presence of nanoparticles, proposing a new nanoFF using less of some additives. The behavior of both, the original and optimized nanoFF, were tested through dynamic reservoir conditions tests. Finally, the first worldwide field application of this kind of fluid was made in the Castilla oil field, with remarkable results. Just as described, the results are also presented in the same order.

### 3.1. Nanoparticles Characterization and Selection of the Best according to Their Capacity of Enhancing HO Mobility

#### 3.1.1. Nanoparticles Characterization

**Figure 2:**
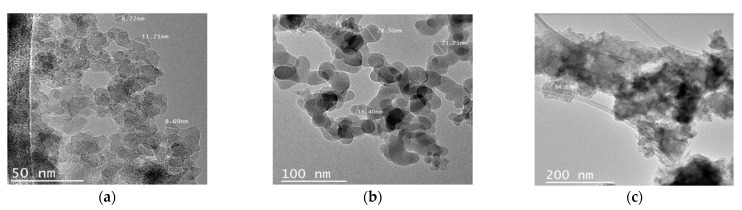
HR-TEM micrographs of (**a**) Si07, (**b**) Si200 and (**c**) Al nanoparticles.

Figure 2 shows the TEM micrographs of Si07, Si200 and Al. It can be noticed that all samples were amorphous, and the particle size followed the order Si07 < Si200 < Al.

The summary of the characterization of the virgin and modified nanoparticles is presented in Table 3. As reported in various works [24,27], particle size is little affected for the surface modification, maintaining the same order as observed for virgin nanomaterials. On the other hand, for all nanoparticle’s chemical natures, a reduction in the surface area can be seen due to surface modification. The basification process is the one that showed to have the more significant impact on this property, reducing the S_BET_ by 44.2, 40.0 and 26.6% for Si07, Si200 and Al, respectively. These results agree with those reported by other authors who also noted that surface modification affects the total acidity of all the samples according to if it is acidification or basification process [27,31]. The values of the isoelectric point, obtained from the zeta potential tests, change depending on the chemical nature of the nanoparticles. For silica can be found at Ph = 2 and for alumina at pH above 6 and below 9.

#### 3.1.2. Crude Oil Viscosity Reduction

**Figure 3:**
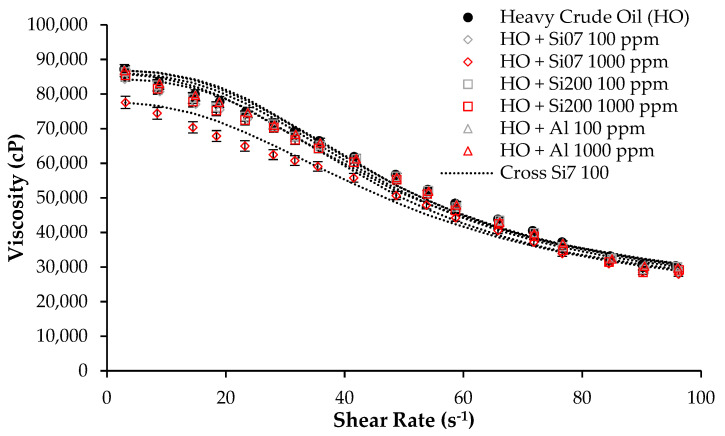
Viscosity as a function of shear rate at 25 °C for HO in the absence and presence of Si07, Si200 and Al nanoparticles at 100 and 1000 mg/L and fitted with the Cross rheological model.

Figure 3 shows the viscosity at different shear rates for the HO, and for this, when adding each unmodified nanoparticle at two concentrations: 100 mg/L and 1000 mg/L. These concentrations used are selected based on our previous work, which have been the most representative for altering the wettability and the viscosity of the heavy and extra-heavy crude oil reservoirs [7,46].

On the other hand, Table 4 presents the estimated parameters of the Cross rheological model adjusted for each plot and the viscosity reduction, with RSME% values lower than 5%. In these tests, carried on at room temperature, the first result that can be noticed is related to the pseudo-plastic or shear-thinning behavior of the HO, which means that its viscosity reduces as the shear rate increases, which is widely reported for heavy crude oils [7,46]. There was also observed that the effect of the lowest dosage of any nanomaterial was null. Indeed, when adding 1000 mg/L of nanoparticles, only Si07 propitiates a reduction in the HO viscosity for all the evaluated shear rates. Precisely, the estimated value for *µ_(0,__γ)_* parameter of the Cross model, related with the viscosity behavior of the HO at a near-zero shear rate, supports that conclusion, being reduced nearly by 11.65%. This is similar to the 10.95% value obtained for the calculated %DVR at 10 s^−1^. This behavior can be explained due to interactions between the hydroxyl bonds of asphaltenes compound, characteristic of the heavy crude oils and the silanol groups present on the surface of silica nanoparticles [47].

**Figure 4:**
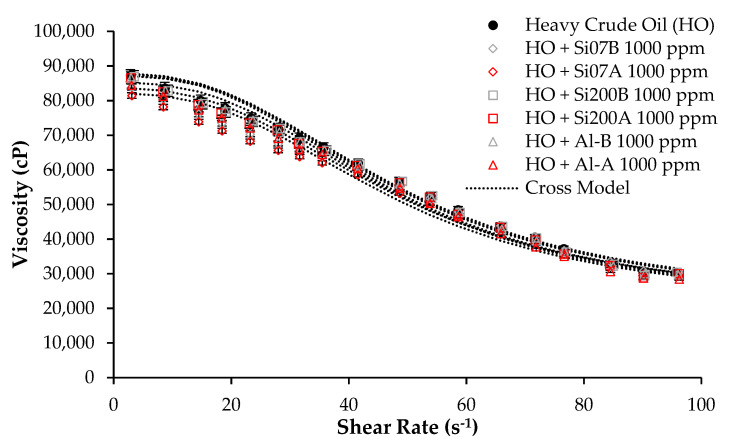
Viscosity as a function of shear rate at 25 °C for HO in the absence and presence of Si07, Si200 and Al nanoparticles after surface modification processes at 1000 mg/L and fitted with the Cross rheological model.

Considering the previous result, evaluating the impact of the surface modification processes over the rheological properties of the HO was carried out using 1000 mg/L of the nanomaterials. Figure 4 and Table 5 present the viscosity at different shear rates and the estimated parameters for the adjusted Cross model for all the measures. To summarize, it can be noticed for all nanoparticles that surface modification has no significant effect on the rheological properties of the HO. In fact, for Si07 can be seen that its potential of reducing crude oil viscosity slightly decreases with the acidification process and in major magnitude with the basification process.

At this point can be noticed the trends reported by Taborda et al., who found for silica nanoparticles a direct relationship between the particle size and the DVR% [48]. In this sense, at nanoparticles dosage of 1000 mg/L, the degree of viscosity reductions follows the order Si07 > Si07A > Si07B > Si200 > Si200A > Si200B. In this sense, as the authors noticed [48], for each size of a silica nanoparticle, the nanomaterials without surface modification presented the highest DVR% due to the presence of silanol groups and the highest surface area values compared to acid and basic modification [48]. On the other hand, alumina nanoparticles did not show a trend related to the DVR% with their particle diameter or surface area.

#### 3.1.3. Nanoparticle Wettability Alteration

**Figure 5:**
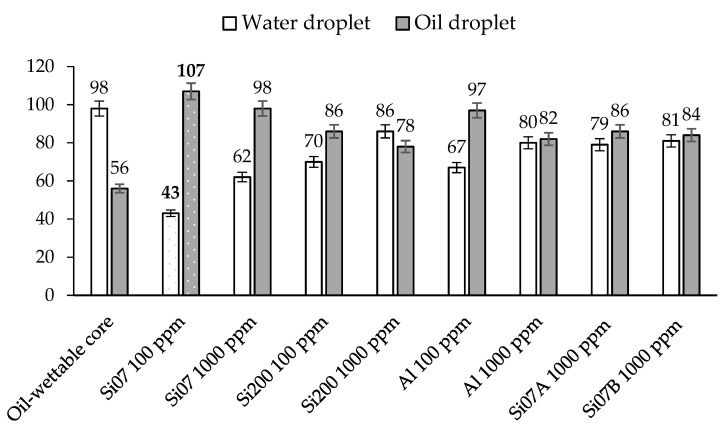
Contact angles to oil and water measured at 25 °C for oil-wettable systems before and after being treated with aqueous dispersions of Si07, Si200 and Al at 100 and 1000 ppm at 77 °C for 24 h.

**Figure 6:**
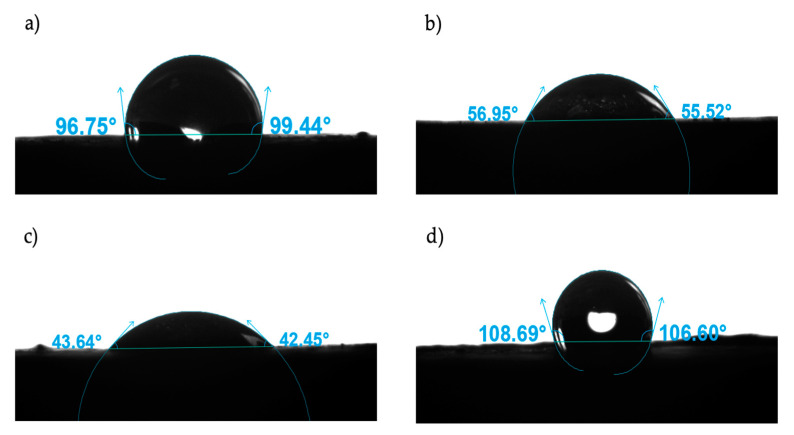
Contact angles to (**a**) water and (**b**) oil for an oil-wettable system, and for (**c**) water and (**d**) oil for an oil-wettable system after being treated with aqueous dispersions of Si07 at 100 ppm at 77 °C for 24 h. All the measures were made at 25 °C.

Figure 5 presents the contact angles for water/air/rock and oil/air/rock systems at 25 °C. It can be seen for all the oil-wettable rock systems treated with different types of nanoparticles that the contact angle for the water droplet decreased as the oil droplet angle increased. As shown in Figure 6, the best result was obtained with Si07 at 100 mg/L, improving the water wettability by 56% regarding the base system and reducing the oil wettability, which can be seen in the increment of the oil droplet angle from 56° to 107°. This nanomaterial also showed the best behavior for concentrations of 1000 mg/L, reaching a 37% more water-wettable system. The better results obtained for the lowest concentration of nanoparticles agreed with those reported by other authors, even related to fracturing fluid applications [22,27]. There can also be noticed that the nanoparticle surface modification processes did not improve the wettability alteration of the rock. However, for the rocks treated with Si07 and Si200 can be noticed that there is a direct relationship between the particle size, and hence its surface area, with the water droplet contact angle. These results can be explained by the major amount of active sites available in the nanoparticles with the highest surface area, which propitiates better adsorption of the asphaltenes, removing those of the porous media and making it less oil-wettable [22,24].

### 3.2. Static Tests of Adding Nanoparticles to a Commercial FF

Static tests were carried out to the commercial LG in the presence and absence of the ZPM. Assessing this scenario was necessary due to this additive’s effect over the proppant, clumping the grains. After that, the next step was to evaluate the FF with its complete formulation and the role of adding nanoparticles in that kind of complex fluid.

#### 3.2.1. Linear Gel

**Figure 7:**
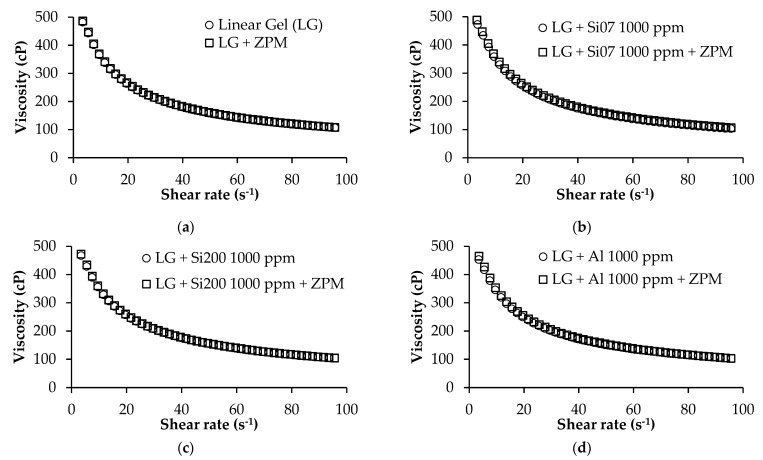
Viscosity as a function of shear rate at 25 °C for LG with and without (**a**) ZPM at its dosage in the FF formulation in the absence and presence of (**b**) Si07, (**c**) Si200 and (**d**) Al nanoparticles at 1000 mg/L.

As previously mentioned, the commercial FF formulation has the particularity of containing a ZPM to agglomerate the proppant in the fracture to disfavor its displacement in the flowback stage. Thus, it was necessary to guarantee that this phenomenon does not happen to the nanoparticles when added to the FF, and it was evaluated through rheological tests of the linear gel phase. This assessment allowed us to observe if there can be seen some effect in LG viscosity attributable to agglomeration or instability of the evaluated nanoparticles. Figure 7a–d shows the rheological behavior of linear gel with and without different nanoparticles before and after adding ZPM. All the measures were carried on at 25 °C. The viscosity of linear gel did not change in any of the evaluated scenarios; therefore, the stability of each nanoparticle is demonstrated before and after being in contact with the ZPM. In addition, it was observed for the LG in all tests a pseudo-plastic behavior that decreased its viscosity as the shear rate increased [49]. This is characteristic of this type of polymeric fluids [50] due to the larger guar gum molecules [51] than molecules such as water that possess Newtonian rheological behaviors. Finally, the surface-modified nanoparticles were not tested due to their behavior in the crude oil rheology and wettability alteration test.

#### 3.2.2. FF and Interactions Polymer-Nanoparticles

Table 6 presents the results of evaluating the original FF with and without adding the different commercial nanoparticles at 1000 mg/L. These tests were carried on to emulate the first effort of the test API RP39 but at 25 °C and atmospheric pressure. The main goal of this methodological proposal is to enable a quicker and cheaper way to reach valid conclusions in shorter times with lower costs than those observed in the typical high-pressure tests. The results showed that none of the evaluated nanoparticles deteriorated the desired rheological behavior of the fracturing fluid. On the contrary, they improve it.

**Figure 8:**
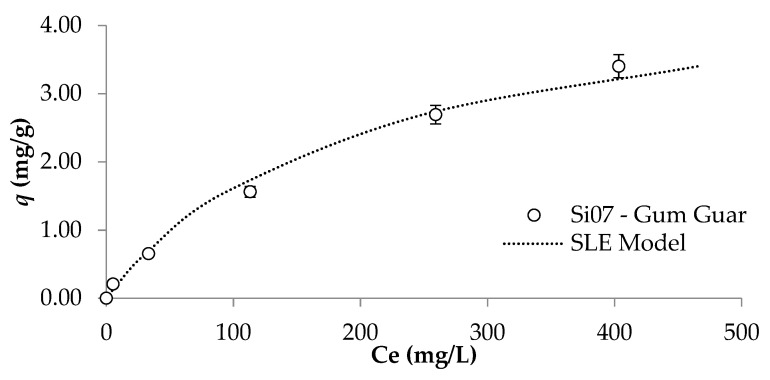
Adsorption isotherm of guar gum polymer onto Si07 nanoparticles at 1000 mg/L at a temperature of 25 °C and atmospheric pressure.

Considering the previous results and those related to the crude oil viscosity reduction and wettability alteration, there was chosen the nanoparticle Si07 at a dosage of 1000 mg/L to improve the commercial FF and for further assessments. In this sense, it is crucial to understand the interactions between the gum guar-based polymer and the nanoparticles. Thus, Figure 8 presents the adsorption isotherm between the Si07 nanoparticles fixed at 1000 mg/L and the abovementioned polymer at different concentrations ranging from 1000 mg/L to the one corresponding to the formulation of the FF, 9300 mg/L. This procedure is equivalent to the method I proposed in an our previous work [35]. According to IUPAC classification [52], the adsorption of polymer onto the surface of Si07 nanoparticles presents the shape of an isotherm Type I (b). This is characteristic of an adsorptive process in monolayer, in which the amount adsorbed is approaching a limit value determined by the volume of accessible micropores and narrow mesopores, more than their internal surface area [52]. This type of isotherms is also related to a high affinity between the adsorbent and the adsorbate. All these results are supported by the estimated parameters of the SLE model [44], presented in Table 7 that were adjusted with a good fit, especially by the low value of the *H* parameter, related to Henry’s region and hence to the adsorption affinity. These results agree with Li et al. [53], which found that silica nanoparticles can inhibit the bonding between the guar gum-based polymer. Their work discovered that the polymer auto-association process was carried out through the hydrogen bonds formed by ring-connected hydroxyl groups and free hydroxyl groups. However, in the presence of silica nanoparticles, the last formed hydrogen bonds with the nanomaterials disadvantaging the interaction between the molecules of this kind of polymer. This situation leads that Si07 nanoparticles constitute a sort of nucleation point.

**Figure 9:**
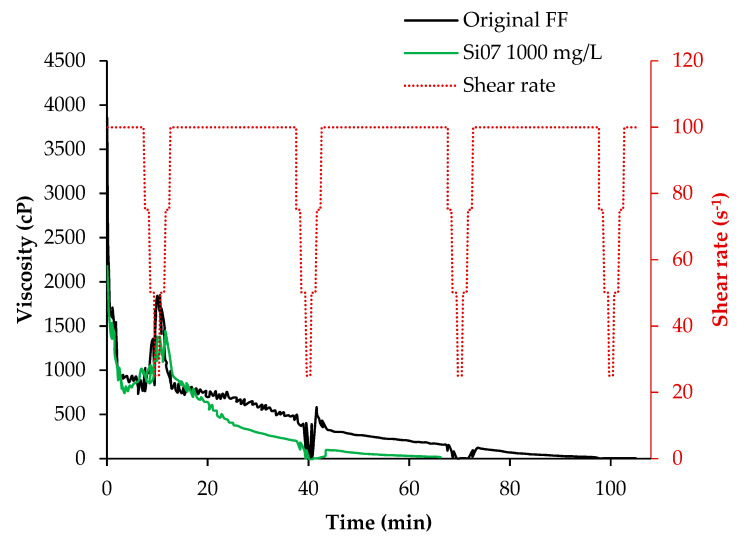
Rheological behavior of the original FF with and without 1000 mg/L of Si07 under pressure and temperature of 87.7 °C and 5500 psig.

Having selected the best nanoparticle and its dosage, Figure 9 presents a complete test of rheological behavior for the fracturing fluid in the absence and presence of Si07 at 1000 mg/L and their rupture, under 5500 psig and 87.7 °C and over a specific time. For the original FF can be seen at the beginning of the test high values of viscosity due to the arming process carried out by the polymer and the crosslinker compounds, presenting a maximum value of 1842 cp at a shear rate of 25 s^−1^ at approximately 10 min. This result is coherent with the data presented in Table 6, where can be noticed similar rheological behaviors, even at the other evaluated shear rates. These results proved that this method could help estimate the typical API RP39 test results at low temperature and pressure. After 40 min of the start of the high-pressure test, for the second effort, the original FF continued showing values of viscosity above 500 cp. However, at the last stage of the trial, this fluid was disarmed by the activation and action of the breakers and showed viscosity values below 100 cP.

On the other hand, the FF containing 1000 ppm of Si07 showed similar behavior to the original in the first effort, but showed a fall in their viscosity measures right after, showing values below 50 cP at the beginning of the second effort. This situation experienced in the presence of silica nanoparticles can be explained by the findings made with the adsorption isotherm of the guar gum-based polymer onto this kind of nanomaterial. In the absence of nanoparticles, the free hydroxyl groups would form a hydrogen bond between them, even with the help of the action of the crosslinker. On the contrary, those free groups would rather interact with the Si07 nanoparticles when added, making them a nucleation point. In the last scenario, fewer hydrogen bonds would be formed by polymer-polymer interactions. Finally, if we consider that usually the breakers added to a fracturing fluid are mainly designed to disrupt this type of interaction, it would be more effective in the presence of Si07 when those happen with less frequency.

#### 3.2.3. Optimized Fracturing Nanofluid

**Figure 10:**
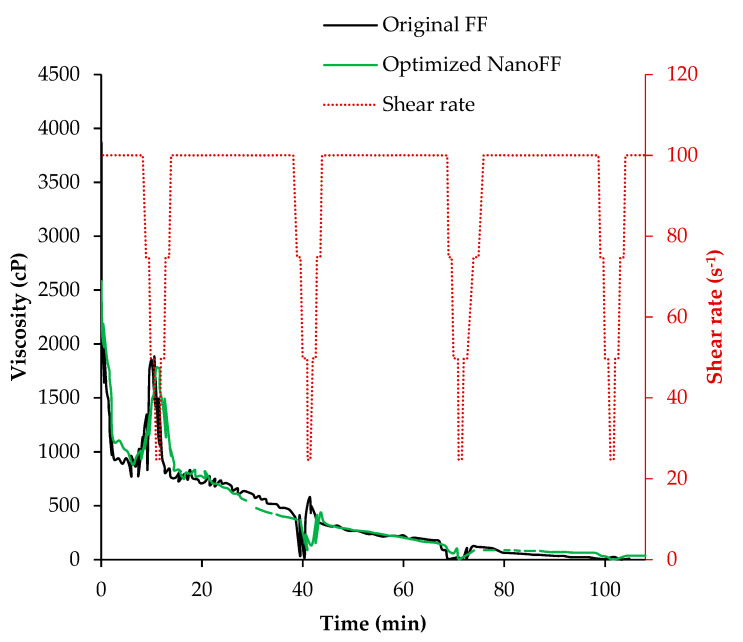
Rheological behavior of the original FF and optimized NanoFF under pressure and temperature of 87.7 °C and 5500 psig.

The previously described situation regarding the interaction between the system nanoparticle-polymer when the breaker additives are activated can be considered an opportunity to optimize the formulation of the original FF with 1000 mg/L of Si07. In this sense, to reach the desired rheological behavior for this FF, it was reduced by 20% the delayed breaker and by 10% the other one used in the FF formulation. Figure 10 presented the high-pressure and high-temperature rheological test of the optimized fracturing nanofluid (Optimized NanoFF), showing its behavior through the evaluated time. It can be noticed that both the original FF and the optimized NanoFF presented the same behavior over all the efforts, evidencing the role of nanotechnology in improving this kind of fluids.

**Figure 11:**
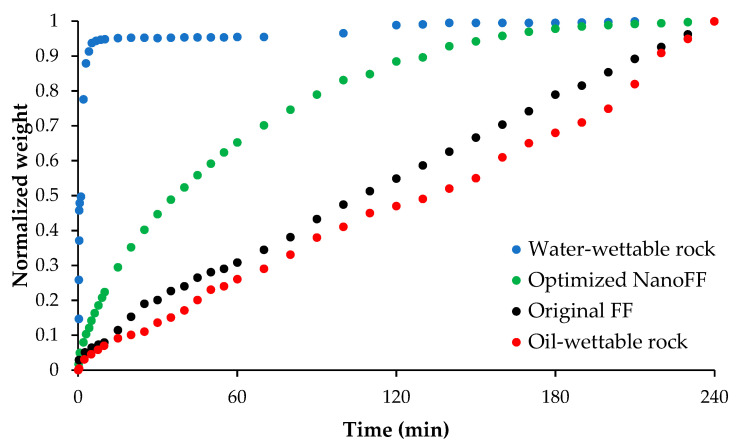
Spontaneous imbibition curves made at 25 °C and atmospheric pressure to the synthetic Ottawa sand cores for the water-wettable and the oil-wettable systems and the last one after contact with the original FF and the optimized NanoFF for 16 h at 87.7 °C and 50 psi.

To original and optimized NanoFF were important to determine the impact of both in the alteration of rock wettability through a quantitative evaluation. Thus, Figure 11 presents the spontaneous imbibition tests carried out to a water-wettable rock, with their oil wettability restored and after being immersed in the assessed fracturing fluids. The obtained results showed that the imbibed water for the original FF system was the closest to the oil-wettable rock. If we consider the hydrophilic nature of the guar gum-based polymers, it is clear the inability of the original FF to make the system more water wettable [27]. On the other hand, for the optimized NanoFF, it is clear the effect of the presence of Si07 nanoparticles at 1000 mg/L, e.g., at 120 min the normalized weight followed the order: oil-wettable rock < original FF < optimized NanoFF < water-wettable rock with values of 0.47, 0.55, 0.88 and 0.99, respectively. These results were supported by works that expose nanoparticles as agents that improve the wettability of sandstones [54,55].

### 3.3. Dynamic Tests Made to Original FF and Optimized NanoFF

#### 3.3.1. Proppant Media Tests

**Figure 12:**
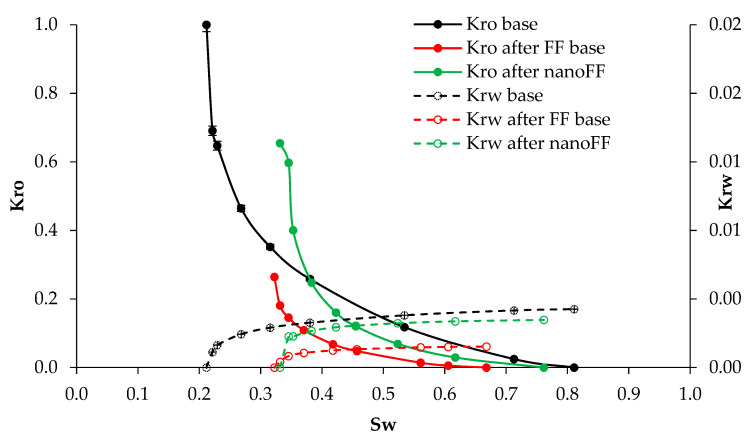
Relative permeability curves before and after injection of the original and optimized NanoFF were conducted in the proppant porous media.

The first pair of dynamic tests were carried out to emulate the conditions created inside the new zone generated by the fracture operation and filled with the proppant. In this sense, Figure 12 presents the relative permeabilities to oil (Kro) and water (Krw) for the base system and the systems with the disarmed original FF and the optimized NanoFF. It can be seen that the presence of any FF causes some damage to the porous media. However, there are remarkable differences between both evaluated fluids. One is related to the increment of 14.3% in the residual oil saturation (Sor) for the original FF, compared with the 5.15% obtained with the optimized NanoFF. This result means that less oil will be retained in the proppant porous media in the fracking operations made with a nanoFF. Regarding the residual saturation of water (Swr), an increment for the system in the presence of nanomaterials can also be noticed, showing a Swr of 11.7%. In comparison, the system with the original FF showed 11%. Thus, the nanoparticles also propitiate the retention of water in the media.

**Figure 13:**
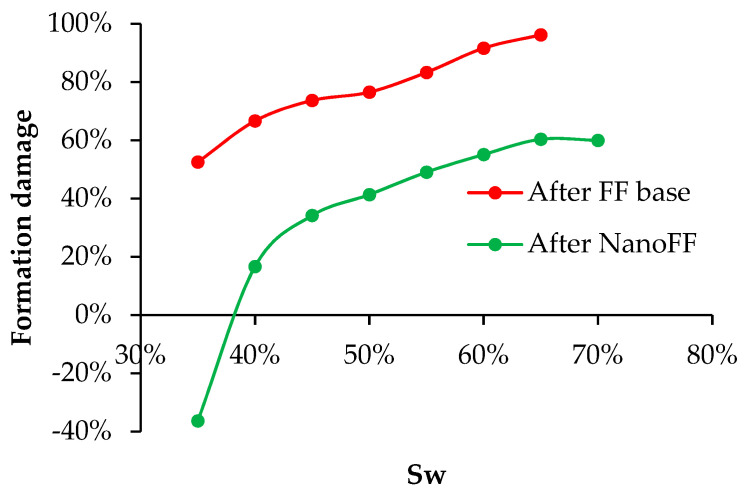
Formation damage behavior at different water saturations for the test carried out with the original and optimized NanoFF in the proppant porous media.

However, the more significant difference in the systems with the absence and presence of nanoparticles can be seen in the behavior of Kro. Hence, Figure 13 presents the graphs of damage, measured as the reduction of the Kro in comparison with the base system, against the water saturation. It was observed that the lowest damage caused by the original FF was 53% for a Swr of 35% and reached a maximum damage value of 96% for a Swr of 65%. On the other hand, in the intervale defined by the start of its mobility window and a Swr of approximately 40%, the system with optimized NanoFF is above the base system, causing a stimulating effect. After that, the formation damage was always much lower than observed for the original FF system. As expected, the reductions in damage led to increments in the recovered oil. Therefore, a 28% higher crude oil recovery factor was observed for the FF in the presence of nanoparticles compared with the fluid without them.

#### 3.3.2. Formation Core Tests

**Figure 14:**
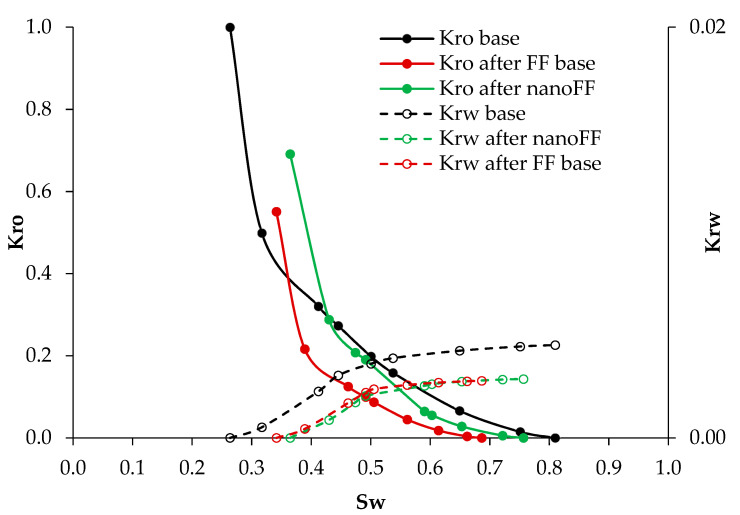
Relative permeability curves before and after injection of the original and optimized NanoFF were conducted in the Castilla oil field porous media.

**Figure 15:**
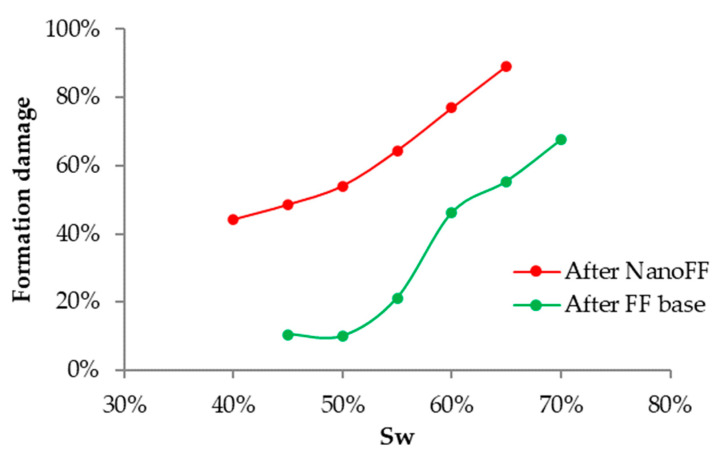
Formation damage behavior at different water saturations for the test carried out with the original and optimized NanoFF in the Castilla oil field porous media.

After the previous test, the next pair of the dynamic test was made in a representative formation core of the Castilla Oil field. The evaluated fracturing fluids are put in contact with the porous media when armed and were let to disarm by the action of the pressure and temperature [35]. Figure 14 presents the relative permeability curves for this test carried out for the original FF and the optimized NanoFF. It can be observed that the reduction of the crude oil mobility was lower in all its mobility window when there was used the optimized NanoFF, remaining even close to the Kro base for Swr from 0.43 to 0.75. The Sor increased by 13.2% without nanoparticles, while the one with those only increased 5.3%. Just as happened with the proppant porous media, the formation sample will retain less oil when using a fracturing fluid improved with nanotechnology. Also, the optimized NanoFF led to an increase in the Swr by 10.1%, compared with the 6.9% caused by the original FF. Figure 15 presents the graphs of damage, measured as the reduction of the Kro compared to the base system, against the water saturation. It was observed that the lowest damage caused by the original FF was 44% for a Swr of 40% and reached a maximum damage value of 89% for a Swr of 65%. On the other hand, for the optimized NanoFF, the formation damage was always much lower than the observed for the original FF system. These results lead to an 18% increment in the recovered oil for the process containing nanomaterials compared with the one without them.

**Figure 16:**
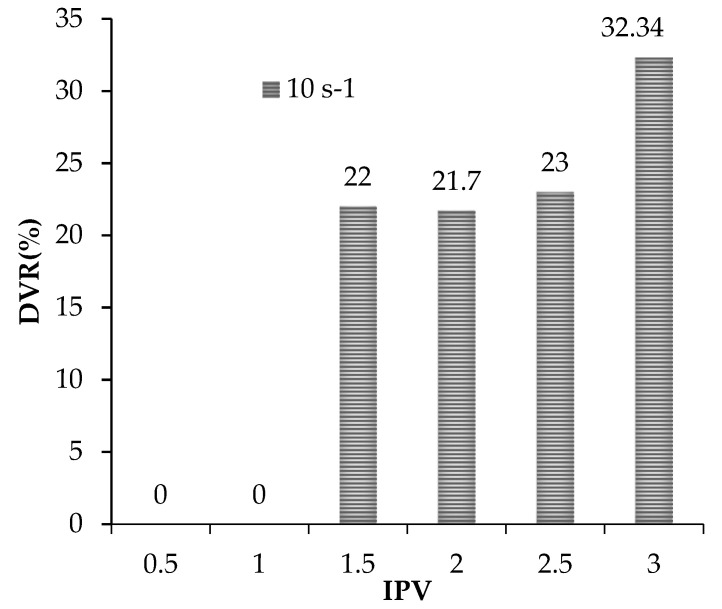
DVR% for the different porous volumes produced in the dynamics tests carried out in the Castilla oil field porous media with original FF and optimized NanoFF.

After each evaluation of the recovery factor in the last dynamic tests, specific porous volumes of oil were injected into the core sample to evaluate the impact of the original FF and the optimized NanoFF in the crude oil viscosity. The oil recovered was collected for each 0.5 injected porous volume (IPV). These oil samples were characterized through their viscosity at different shear rates at 25 °C. Figure 16 presents the viscosity reduction of the HO viscosity compared to the result of the effluents obtained in the absence and presence of Si07 nanoparticles. A 32.3% reduction of the crude oil viscosity produced in the third porous volume from the optimized NanoFF system versus the original FF was observed. There was also noticed that increments in the IPV also cause rises in DVR%. This situation can be explained because as more crude oil flows in the porous media, it is more probable that it interacts with the nanoparticles remaining in it. Viscosity crude oil reduction by interaction with nanoparticles has been reported in several works, relating this phenomenon to adsorptive processes between the surface of the nanoparticles and the asphaltenes present in heavy crude [7]. Precisely, the asphaltenes nature leads them to self-aggregation processes forming large viscoelastic networks with the resins and therefore increasing the viscosity of the crude oil [56]. In this sense, the high affinity between asphaltene and silica-based nanoparticles caused the last to present a high absorption capacity for this type of molecule [42]. This situation diminishes the size of the asphaltene aggregates and disrupts the formed viscoelastic networks generating crude oil viscosity reduction, especially for HO with a high asphaltene content [7].

### 3.4. Field Application in the Castilla Oil Field

**Figure 17:**
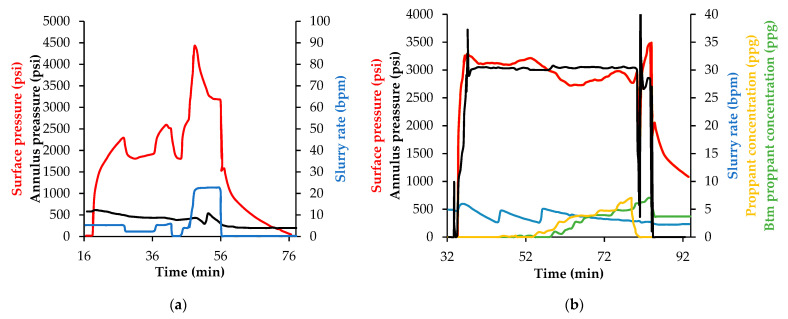
Pressure behavior in the surface, in the annulus and slurry rate at different times while (**a**) opening the fracture and (**b**) injecting the proppant measuring flow rate in surface and the bottom of the well in the Castilla field application.

The first hydraulic fracture operation was carried out in February 2019, initially 126 bbls of optimized NanoFF preflux were pumped during the diagnostic pumping operations to condition the media, and then, after a soaking time, 180 bbls of the main NanoFF were mixed as part of the pad stage of the treatment to place 121,600 lb of proppant material. Figure 17 presented the behavior of surface and annulus pressures, the slurry rate, surface and bottomhole proppant concentration. As shown in Figure 17a, a conditioning schedule was pumped, the DIX was injected to propitiate the dissolution of the organic scale, then, the optimized NanoFF preflux was injected and another stage of DIX was pumped. Finally, as shown in Figure 17b hydraulic fracture was generated, the first portion of the pad (~180 bbls) were containing the optimized NanoFF as part of the schedule that placed the sand that reach values near 8 pounds per gallon (ppg) of concentration.

**Figure 18:**
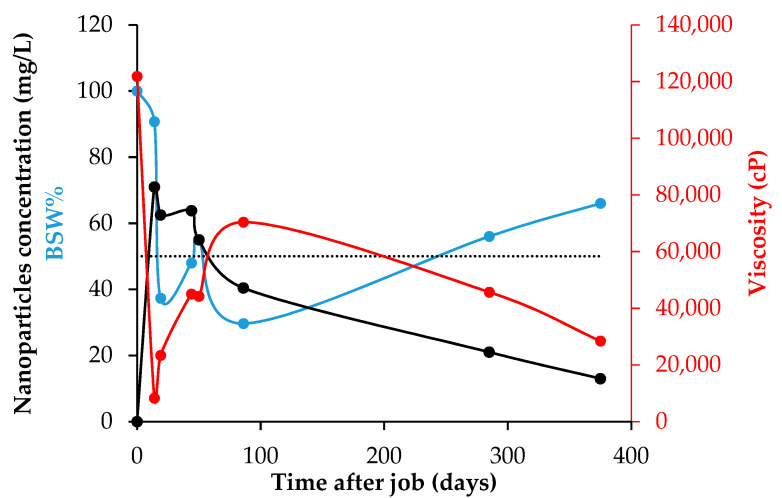
Behavior over time for the viscosity, BSW% residual nanoparticle concentration for the produced oil in the well when was carried out the fracking operation with the optimized NanoFF in the Castilla oil field.

In Figure 17 can be noticed the success of the hydraulic fracture operation, showing the impact in the usage of nanomaterials for improving the formulation of a fracturing fluid reducing additives like the breakers, enhancing the rheological behavior. However, the main benefit of using nanotechnology in this scenario, is the opportunity to improve the production conditions by increasing the mobility ratio of oil/water in the porous media during a sustainable period of time. Figure 18 summarizes the results of the rigorous monitoring made to the HO viscosity, the BSW%, and how they are linked with the residual nanoparticle concentration measured into the produced fluids. For day 0, related to the baseline before the operation, a HO viscosity value of 121,800 cP was obtained with BSW% higher than 98%. Approximately 20 days after making the hydraulic fracture, the crude oil viscosity reached its minimum of 8280 cP, equivalent to 6.8% of its original value. The maximum measured viscosity was 70,367 cP after three months and have passed more than a year of the operation, there was measured 28,440 cP, near a quarter of its original viscosity. The BSW% followed a similar behavior, falling to 37.3% in the first 20 days and reaching its lowest measure after 86 days, 29.7%. Since then, the BSW% slowly increased, having a value of 66% after one year of the fracture operation. This result is highly important when considering that the BSW% was near 100% before this, and the well production was almost water. Finally, all these results were consistent with the measured nanoparticle residual concentrations, which showed values around 65 ppm in the first 6 weeks and decreased at low rates until 13 ppm after 375 days. The consistent reduction in oil viscosity over a period of +460 days shows a perdurability of the effects in the reservoir fluid even higher than the observed during lab testing which has allowed conducting more than twenty jobs of this type where the same effect has been observed. The combined effect of wettability alteration and fluid viscosity decrease result in a positive impact on the Estimated Ultimate Recovery (EUR) of the wells where this technology have been deployed. Indeed, after two years of this field test the accumulative incremental production is around 120,000 oil barrels.

## 4. Conclusions

Commercial nanoparticles were modified in their surface, characterized and tested, determining their capacity to improve the HO mobility produced in a well in the Castilla oil field. All these when added to a commercial fracturing fluid without affecting its rheological behavior. According to contact angle measures, the static tests revealed that water-wettability alteration was favored more than 50% when adding Si07 nanoparticles. Related to viscosity, these nanoparticles at 1000 mg/L can lead to a DVR% greater than 10% at 25 °C. For this reason, this chemical nature of nanomaterial at the abovementioned dosage was selected to improve a commercial FF.

There was proposed a methodology for evaluating the rheological behavior of armed FF at low pressure and temperatures that approximates the more expensive API RP39 test results. Precisely, in this kind of test, there was found that adding 1000 mg/L at the commercial fracturing fluid can lead to reducing its expected viscosity value after the first effort. In this sense, the evaluated adsorption relation between the Si07 and the guar gum-based polymer of the FF formulation revealed an isotherm type I(b) according to the IUPAC classification [53]. There was found a high adsorption affinity between the couple adsorbent and adsorbent. It can be explained by the hydrogen bonds formed between the free hydroxyl groups in the polymer and the silica-based nanoparticle [54]. These results can explain the reduction of both kinds of breakers used in the formulation of the commercial FF by 20 in the delayed one and 10% in the peroxide breaker, proposing an optimized NanoFF.

Dynamic tests were carried out to proppant and Castilla porous media for the original and optimized NanoFF. The Sor increased almost three times for the FF without nanoparticles in the proppant package. The formation damage, measured for the changes in the Kro, was below all the saturation window for the product containing nanoparticles, showing even a stimulation when compared to base conditions in Sw between 35 to 40%. The highest value of formation damage measured was 60% for the NanoFF, while it was 96% for the original FF. A similar situation can be found in the Castilla formation core, but also having a 30% smallest increment in the Swr, which means that adding nanomaterial propitiates water retention in the reservoir compared to the scenario without them. Regarding the formation damage, there was found that the optimized NanoFF presented lower values than optimized FF in all the saturation window with a minimum measure of 10% for the first one and 44% for the last one. The produced crude oil over the dynamic tests showed a reduction of 32.3% in its viscosity related to the presence of Si07 nanoparticles.

Finally, the Castilla field application of the optimized NanoFF was itself the more impressive product of this investigation work. Motivated by the static and dynamic test results, Ecopetrol S.A. made a successful fracking operation with the developed FF, carried out as desired without any setback. Before the intervention, the crude oil viscosity was 121,800 cP and its BSW% approximately 98%. After nearly two weeks of production, the crude oil viscosity fell 93.2% to its minimum value of 8280 cP, and after more than a year was 28,440 cP. The BSW% reached its lowest value at 29.7%, and passing 375 days was nearly 66%. All these results can be related to the presence of nanomaterial, especially to those measured in the well-produced fluids with concentrations above 13 mg/L in the same time window, showing the durability and the potential benefits of nanotechnology applied to the oil & gas industry, generating in this case an incremental of 120,000 oil barrels for only one field test in a period of two years.

## Figures and Tables

**Table 1:** Characteristics of the porous media representative of the reservoir and employed in the dynamic test.

Sample	Core	Carbolite Porous Media
Length (cm)	6.8 ± 0.1	7.2 ± 0.1
Diameter (cm)	3.7 ± 0.1	3.8 ± 0.1
Porosity (%)	21.8 ± 0.2	26.4 ± 0.2
Effective permeability to water (mD)	14.5 ± 0.2	477 ± 3
Effective permeability to oil (mD)	1372 ± 4	13,995 ± 9

**Table 2:** Additives, their orders and dosages in preparing the different fracturing fluids (FF).

Order	Additive	Dosage % *v*/*v*
1	Bactericide	0.005
2	Clay stabilizer	0.200
3	Guar gum-based polymer	0.875
4	Surfactant	0.200
5	Zeta Potential Modifier	1.500
6	pH controller	0.400
7	Delayed breaker	0.400
8	Peroxide breaker	0.100
9	Metaborate crosslinker	480 mg/L

**Table 3:** Estimated mean particle size (d_p-50_), surface area (S_BET_), total acidity and the isoelectric point of the Si07, Si200 and Al after surface modification processes.

Nanoparticle	d_p-50_ ± 0.2 nm	S_BET_ ± 1 m^2^/g	Total Acidity ± 0.02 mmol/g	Isoelectric Point ± 0.03 (pH)
Si07	11.6	388	2.16	2.40
Si07A	12.9	216	2.85	2.05
Si07B	13.4	143	1.29	2.25
Si200	37.2	303	1.99	2.35
Si200A	39.7	182	2.91	1.95
Si200B	43.1	106	1.46	2.05
Al	95.0	103	0.61	9.70
Al-A	97.2	76	0.77	6.42
Al-B	97.4	62	0.59	7.38

**Table 4:** Estimated parameters of the Cross rheological models for crude oil in the presence of Si07, Si200 and Al nanoparticles at 100 and 1000 mg/L and 25 °C.

Parameter	HO	Si07 100 ppm	Si07 1000 ppm	Si200 100 ppm	Si200 1000 ppm	Al 100 ppm	Al 1000 ppm
*µ_(∞,γ)_* (cP)	21,497.76	20,953.69	18,617.21	21,884.76	21,157.80	21,701.18	21,698.76
*µ_(0,γ)_* (cP)	87,857.79	85,926.09	77,621.58	86,881.78	86,044.27	86,847.72	84,335.92
*α_c_* (s)	0.0205	0.0213	0.0202	0.0209	0.0210	0.0209	0.0217
*m*	2.55	2.36	2.33	2.61	2.61	2.65	2.67
*RSME%*	3.74	4.78	4.13	4.88	4.86	4.38	4.47
DVR% (@ 10 s^−1^)	-	3.41	10.95	1.24	2.42	0.26	0.61

**Table 5:** Estimated parameters of the Cross rheological models for crude oil in the presence of Si07, Si200 and Al nanoparticles after surface modification processes at 1000 mg/L and 25 °C.

Parameter	HO	Si07B 1000 ppm	Si07A 1000 ppm	Si200B 1000 ppm	Si200A 1000 ppm	Al-B 1000 ppm	Al-A 1000 ppm
*µ_(∞,γ)_* (cP)	21,497.76	20,901.40	20,519.24	21,843.35	21,331.19	20,486.17	21,557.05
*µ_(0,γ)_* (cP)	87,857.79	83,590.54	82,053.06	87,101.90	87,267.08	85,215.54	87,697.57
*α_c_* (s)	0.0205	0.0206	0.0207	0.0205	0.0211	0.0207	0.0208
*m*	2.55	2.56	2.60	2.63	2.61	2.62	2.57
*RSME%*	3.74	3.63	4.08	4.46	4.92	4.37	4.01
DVR% (@ 10 s^−1^)	-	3.69	6.50	0.89	1.27	0.81	2.76

**Table 6:** Rheological measures of the original FF in the absence and presence of Si07, Si200 and Al at 1000 mg/L at 25 °C and atmospheric pressure at 25, 50, 75 and 100 s^−1^.

Shear Rate (s^−1^)	µ FF (cP)	µ FF + Si07 (cP)	DVR%	µ FF + Si200 (cP)	DVR%	µ FF + Al (cP)	DVR%
25	1850 ± 2	2406 ± 3	30.1	3453 ± 3	86.6	2376 ± 3	28.4
50	1332 ± 2	1327 ± 2	0.4	1611 ± 3	20.9	1521 ± 2	14.2
75	998 ± 1	1 078 ± 2	8.0	1184 ± 2	18.6	1260 ± 2	26.3
100	915 ± 1	913 ± 1	−0.2	954 ± 1	4.2	955 ± 1	4.4

**Table 7:** Parameters estimated from the SLE model for guar gum polymer onto Si07 nanoparticles at 1000 mg/L at 25 °C and atmospheric pressure.

*H* (mg/m^2^)	*K* (g/g)	*q* (mg/g)	RSME%
0.037	0.072	5.75	3.99

## Data Availability

The data presented in this study are available on request from the corresponding author. The data are not publicly available due to confidentiality restriction.
